# Deep Learning: Individual Maize Segmentation From Terrestrial Lidar Data Using Faster R-CNN and Regional Growth Algorithms

**DOI:** 10.3389/fpls.2018.00866

**Published:** 2018-06-22

**Authors:** Shichao Jin, Yanjun Su, Shang Gao, Fangfang Wu, Tianyu Hu, Jin Liu, Wenkai Li, Dingchang Wang, Shaojiang Chen, Yuanxi Jiang, Shuxin Pang, Qinghua Guo

**Affiliations:** ^1^State Key Laboratory of Vegetation and Environmental Change, Institute of Botany, Chinese Academy of Sciences, Beijing, China; ^2^University of Chinese Academy of Sciences, Beijing, China; ^3^Guangdong Provincial Key Laboratory of Urbanization and Geo-simulation, School of Geography and Planning, Sun Yat-sen University, Guangzhou, China; ^4^National Maize Improvement Center of China, China Agricultural University, Beijing, China; ^5^Urban Construction School, Beijing City University, Beijing, China

**Keywords:** deep learning, detection, classification, segmentation, phenotype, Lidar (light detection and ranging)

## Abstract

The rapid development of light detection and ranging (Lidar) provides a promising way to obtain three-dimensional (3D) phenotype traits with its high ability of recording accurate 3D laser points. Recently, Lidar has been widely used to obtain phenotype data in the greenhouse and field with along other sensors. Individual maize segmentation is the prerequisite for high throughput phenotype data extraction at individual crop or leaf level, which is still a huge challenge. Deep learning, a state-of-the-art machine learning method, has shown high performance in object detection, classification, and segmentation. In this study, we proposed a method to combine deep leaning and regional growth algorithms to segment individual maize from terrestrial Lidar data. The scanned 3D points of the training site were sliced row and row with a fixed 3D window. Points within the window were compressed into deep images, which were used to train the Faster R-CNN (region-based convolutional neural network) model to learn the ability of detecting maize stem. Three sites of different planting densities were used to test the method. Each site was also sliced into many 3D windows, and the testing deep images were generated. The detected stem in the testing images can be mapped into 3D points, which were used as seed points for the regional growth algorithm to grow individual maize from bottom to up. The results showed that the method combing deep leaning and regional growth algorithms was promising in individual maize segmentation, and the values of r, p, and F of the three testing sites with different planting density were all over 0.9. Moreover, the height of the truly segmented maize was highly correlated to the manually measured height (*R*^2^> 0.9). This work shows the possibility of using deep leaning to solve the individual maize segmentation problem from Lidar data.

## Introduction

During the 20th century alone, the human population has grown from 1.65 billion to 6 billion according to the United Nations^1^. By the middle of the 21st century, the global population will reach up to 9–10 billion (Cohen, 2003; Godfray et al., 2010). The growing population and declining area of cultivated land under the background of global climate change has brought unprecedented pressure on world food production and livelihood security of farmers (Dempewolf et al., 2010; Godfray et al., 2010; Finger, 2011; Tilman et al., 2011). To meet the challenge, developing new methods of crop breeding to increase crop yields is a promising option (Ray et al., 2013).

Traditional crop breeding, such as hybrid breeding, relies on the breeding experience of breeders, which has the disadvantage of long cycle, low efficiency and great uncertainty. With the development of molecular biology technology, especially the next-generation sequencing (NGS) technologies, molecular breeding has revolutionized traditional agricultural breeding into a new era with characteristics of rapid, accurate, and stable (Perez-de-Castro et al., 2012). However, genomics research cannot achieve satisfactory outcome in genetic improvement of complex quantitative traits which is controlled by both biotic and abiotic factors (Kumar et al., 2015). The major reason is the lack of precise and high throughput phenotype data to assist gene discovery, identification, and selection (Rahaman et al., 2015).

In the past, phenotype data was mainly obtained by manual measurement in the field, which was time consuming, labor intensive, and low accuracy. With the demand of precise agriculture and development of remote sensing, image-based method has been successfully applied in obtaining phenotypic data related to plant structure and physiology. For example, Ludovisi et al. (2017) studied the response of black poplar (*Populus nigra* L.) to drought using phenotype data obtained from unmanned aerial vehicles (UAVs) based thermal images; Grift et al. (2011) measured the root complexity (fractal dimension) and root top angle using root images. However, 2D images were sensitive to illumination and lack of spatial and volumetric information, which is more closely related to plant function and yield related traits (Yang et al., 2013). Although stereo-imaging method has been used in some studies to obtain canopy three-dimensional (3D) structure and leaf traits, the overlap of leaves is still a challenge for 3D reconstruction (Biskup et al., 2007; Xiong et al., 2017b).

Light detection and ranging (Lidar), an active remote sensing technology by recording the time delay between laser transmitter and receiver to calculate the distance between the sensor and target, can provide highly accurate 3D information, which has been widely used in the field of forest ecology with its high penetration ability in the past serval decades (Blair et al., 1999; Lefsky et al., 2002; Guo et al., 2014). Recently, it has drawn extensive attention in the field of plant phenotype (Lin, 2015; Guo et al., 2017). For example, Hosoi and Omasa (2012) used the high-resolution portable Lidar to estimate the vertical plant area density; Madec et al. (2017) used ground Lidar to estimate plant height, and proved its advantage over the 3D points derived from the UAV images. These Lidar based methods focus on the parameters acquisition of group level rather than individual crop level, which cannot fully meet the needs of precision phenotypic traits extraction. The bottleneck in individual crop level lies in how to achieve the accurate individual crop segmentation with 3D points.

Currently, there are mainly two kinds of individual object segmentation algorithms for Lidar points, which are widely used in the forest field, i.e., CHM (canopy height model)-based (Hyyppa et al., 2001; Jing et al., 2012) and direct point-based methods (Li et al., 2012; Lu et al., 2014; Tao et al., 2015). The CHM-based methods use the raster image interpolated from Lidar points to depict the top of the forest canopy (Li et al., 2012), which may have inherent spatial error when interpolated gridded height model from 3D points (Guo et al., 2010). Also, CHM-based methods are not suitable for homogenous, interlocked, and blocked canopy (Koch et al., 2006). These limitations can be overcome by point-based methods, such as voxel space projection (Wang et al., 2008), normalized cut (Yao et al., 2012), adaptive multiscale filter (Lee et al., 2010), and regional growth method (Li et al., 2012; Tao et al., 2015). Among them, regional growth method has shown better performance in both confiner and broadleaf forest. For example, Li et al. (2012) developed a regional growth point cloud segmentation (PCS) method from top to down for confiner forests. In broadleaf forest, Tao et al. (2015) proposed a comparative shortest-path (CSP) algorithm from down to top using the terrestrial and mobile Lidar. The regional growth algorithm depends on the choice of seed points. However, in Lidar-scanned maize group, seed points cannot simply use the maximum value within a certain range because the maximum point of an individual maize is often not the center of the maize. Meanwhile, using clustering method (e.g., k-means, density-based spatial clustering of applications with noise) to detect the short and thin stem of maize is also impracticable.

Deep learning, as a new area of machine learning, has strong ability in extracting complex structural information from high dimensional and massive data (Lecun et al., 2015), which has achieved remarkable results in text categorization (Conneau et al., 2017), speech recognition (Ravanelli, 2017), image detection (Sermanet et al., 2013; Song and Xiao, 2016), and video analysis (Wang and Sng, 2015). Convolution neural network (CNN), as the most commonly used method of deep learning, has made state-of-the-art performance in some image-based phenotyping tasks (Pound et al., 2017; Ubbens and Stavness, 2017). For example, Mohanty et al. (2016) used deep learning method to detect plant disease; Xiong et al. (2017a) used CNN to segment rice panicles successfully based on images; Baweja et al. (2018) used deep learning method to count plant stalk and calculated the stalk width. By contrast, CNNs for 3D analysis, especially for 3D object detection, have rarely been found in the phenotypic field.

Currently, 3D CNN structure mainly includes voxel-based method, octree-based method, multi-surface-based method, multi-view method, and direct point-cloud-based method. Each method has its own advantages and disadvantages. The voxel-based method can effectively preserve the spatial relationship between voxels, but it is computationally intensive with massive point cloud data (Maturana and Scherer, 2015; Milletari et al., 2016); octree-based methods have high indexing efficiency but still occupied large storage (Wang et al., 2017); multi-surface based methods require only smooth surfaces as input but are extremely sensitive to noise and deformation (Masci et al., 2015). The method based on multi-view is generally used in individual object classification, which is less computational but difficult to determine the viewing angle to achieve the best identification (Su et al., 2015). The point-cloud-based method cannot fully consider the spatial structure of the point clouds and does not converge easily (Qi et al., 2016, 2017). Moreover, these 3D convolutional neural networks are mainly used in small object tasks because of the high cost of computing memory and time. In all, the direct 3D object segmentation or detection with massive Lidar points in large maize field is still a big challenge.

This study proposed an indirectly way of 3D object detection and segmentation, which used 2D Faster R-CNN (region-based CNN) to detect object in 2D images compressed from 3D points. After that, the detected object in 2D images was mapped to 3D points, which was further used as seed points of regional growth method proposed by Tao et al. (2015). This method fully utilized the 2D CNN method to avoid the high cost of computation and storage of 3D CNNs, and the regional growth method can keep the geometric relationship of individual maize.

## Materials and Methods

### Data

The experiment was conducted in a maize field in China Agriculture University in June 2017. The maize species is ZD958, which is a low-nitrogen-efficient maize hybrid (Han et al., 2015). The area of the field is around 300 m^2^, with a 0.5 m spacing between rows and a 0.2 m spacing within rows, respectively. The maize was scanned using a high-resolution portable terrestrial Lidar (FARO Focus^3D^ X 330 HDR). The size of the sensor is 240 mm × 200 mm × 100 mm and the weight is 5.2 kg. The detection range is 0.6–130 m with large field of view (horizontal: 360°; vertical: 300°). The pulse rate and maximum scanning rate are 244 kHz and 97 Hz, respectively. The Lidar sensor is equipped with good angular accuracy (horizontal: 0.009°; vertical: 0.009°) and scanning accuracy of 0.3 mm @10 m @90% reflectance (**Table 1**).

**Table 1:** The information of the sensor used in this study.

Sensor	FARO Focus^3D^ X 330 HDR
Laser wavelength (nm)	1550
Laser beam divergence (mrad)	0.19
Field of view (°)	Horizontal: 360°; Vertical: 300°
Angular resolution (°)	Horizontal: 0.009°; Vertical: 0.009°
Detection range (m)	0.6–130 m indoor or outdoor with upright incidence to a 90% reflective surface
Pulse rate (kHz)	244
Maximum scanning rate (Hz)	97
Scanning accuracy	0.3 mm @ 10 m @ 90% reflectance
Scanner weight (kg)	5.2
Dimensions (mm)	240 × 200 × 100
Laser class	Laser class 1
Beam diameter at exit (mm)	2.25

**FIGURE 1:**
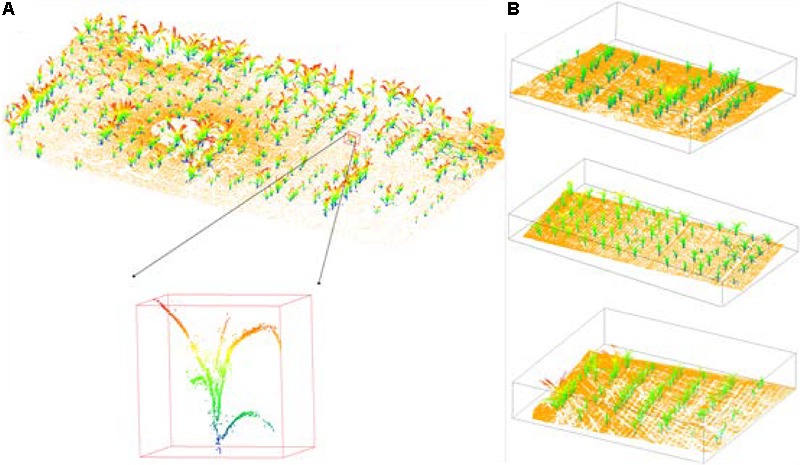
The maize data scanned by terrestrial Lidar of training site and testing sites, whose ground points are in yellow. **(A)** The training site: each maize was manually segmented from the group and colorized by height. **(B)** The testing sites with sparse (top), moderate (middle), and dense (bottom) planting density.

In this study, we collected nine Lidar scans in total. Through the multi-station scanning and registration in FARO SCENE software, we obtained the fully covered Lidar data of the study area. There were around 1000 maize plants of different sizes and shapes in the field. Four sites were chosen to conduct this experiment, one for training, and the other three for testing (**Figure 1**).

#### Training Data (From 3D Points to 2D Images)

**FIGURE 2:**
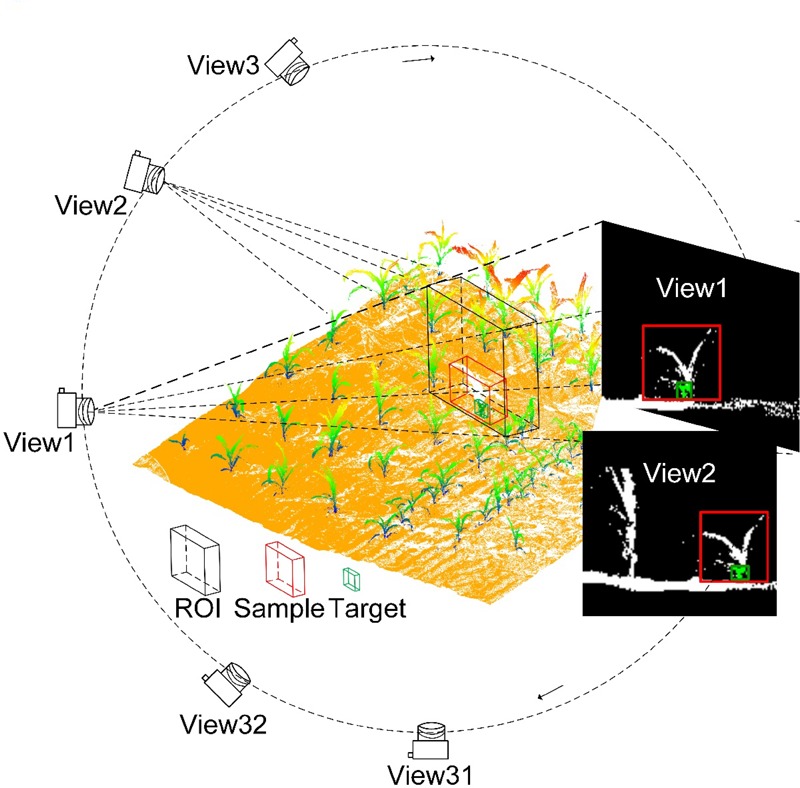
The training samples (deep images) generated through compressing the point cloud within the ROI (region of interest). The field of ROI is 1.028 m × 1.028 m and depth is determined by the depth of each training maize. The ROI covered an individual maize sample segmented manually, whose color is white with a red bounding box. Meanwhile, the target stem of the maize is in green with a green bounding box. Each sample was viewed from 32 different directions, which obtained 32 deep images with different background information. These deep images were used to train the deep convolutional neural network.

In this section, we intended to detect the stem of individual maize with different situations. Because individual maize is always intersecting, it is impossible to directly predict the irregular bounding box in 2D images. However, the stem of maize is always isolated, which is relatively easy to detect from the 3D point cloud data and can be used as the seed point for individual maize segmentation. We prepared different stem samples of individual maize with different background information. In this study, the training site was planted with a density of 3.35 plants/m^2^, whose area was 100 m^2^ (**Table 2**). We segmented the individual maize from the training group to obtain 337 individual maize samples manually using the Green Valley International^®^ LiDAR360 software, which was used to generate the training samples (**Figure 1A**). If we viewed the individual maize from different directions, the background information was different, which was also determined by the field and depth of the region of interest (ROI). For each individual maize in the training group, we viewed from 32 different directions through rotating the group data at a fixed angle separate of 360°/32. The ROI of each view had a field of 1.024 m × 1.024 m, and depth of 0.256 m, which was enough to cover individual maize of our study (**Figure 2**). The fixed size had the effects like data normalization, which was beneficial for speeding up the training process of finding the best solution when using gradient descent as well as promoting the testing accuracy of the neural network. The black bounding box of ROI contained the individual maize with a red bounding box, and its corresponding stem. The points whose z values were smaller than 1/3 of the maize height were chosen as points of a target stem, which was colorized in green with a green bounding box. The ROI filled with maize and stem was compressed into a depth image.

**Table 2:** The dataset information of the training site and the three testing sites of different planting densities.

		Number of maize	Area (m^2^)	Plant density (plants/m^2^)	Maize height (m)		
					Minimum	Mean	Maximum
Training data		337	100.48	3.35	0.09	0.34	0.68
Testing data	Sparse	62	23.05	2.69	0.13	0.32	0.49
	Moderate	71	11.48	6.19	0.13	0.29	0.69
	Dense	88	9.81	8.96	0.14	0.35	0.73

Through multi viewing and compressing, we generated 10784 training images with targets in various backgrounds. The massive training samples were used to train the deep CNN, which helped to learn different features and avoid over-fitting.

#### Testing Data

Three sites with planting density from sparse to dense were used to test the accuracy and robustness of the method. The sparse site was planted with a planting density of 2.69 plants/m^2^ in an area of 23 m^2^, with the maize height ranging from 0.13 to 0.49 m. Maize in this site have big interval, but there were overlaps if we viewed from top. The moderate site had a planting density of 6.19 plants/m^2^ in an area of 11.48 m^2^, with the maize height ranging from 0.29 to 0.69 m. Maize in this site is very homogeneous with small intervals. The dense site was planted with a planting density of 8.96 plants/m^2^ in an area of 10 m^2^ with the maize height ranging from 0.14 to 0.73 m. Maize in the dense site were heavily intersected and overlapped. In addition, the number of maize of the three sites were 62, 71, and 88, respectively (**Table 2**).

### Methods

#### Faster R-CNN Model

Faster R-CNN (Ren et al., 2015) was a major breakthrough in the field of target detection with images using deep learning method after R-CNN (Girshick et al., 2014) and Fast R-CNN (Girshick, 2015). R-CNN was slow, because it ran the CNN around 2000 times (region proposals generated by selective search method) per image and had to train three different models separately, one for extracting image features, one for classifying bounding box, and one for regression to tighten the bounding box. Fast R-CNN enhanced these deficiencies by jointing the feature extractor, classifier, and regressor in a unified framework, which ran the CNN just once per image and then shared the feature images across the ∼2000 region proposals. Even though, Fast R-CNN still used the selective search method to create proposals, which is the bottleneck of the overall process. Faster R-CNN replaced the selective search with a regional proposal network (RPN), which reused the feature maps of CNN. This “end-to-end” framework can be thought as a combination of RPN and Fast R-CNN. Feature parameters were shared between the two networks.

**FIGURE 3:**
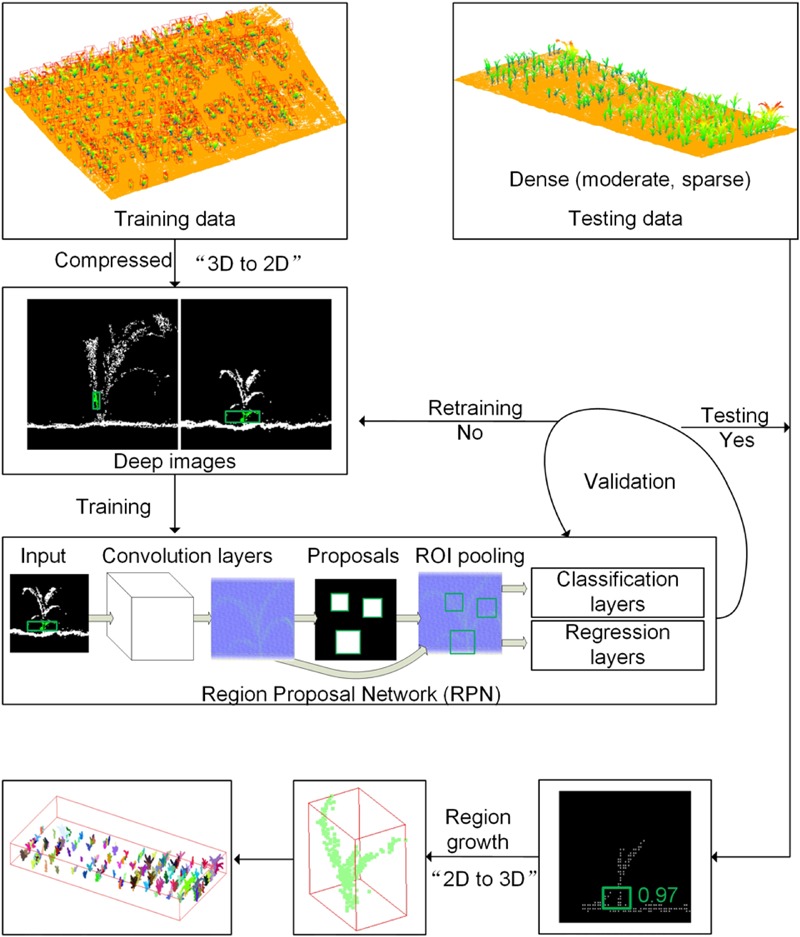
The flow chart of the Faster R-CNN to detect stem in deep images and the regional growth method for individual maize segmentation. Each training maize was compressed into deep images and fed into the Faster R-CNN to learn the ability of classifying and regressing the anchor (bounding box) of the stem in deep images. The testing sites were sliced row by row to generate deep images, which were tested to get the anchor of stem by the trained Faster R-CNN. The detected anchor was mapped to three dimensional points, which was used as seed points to grow an individual maize from bottom to up.

In this study, each 2D view from 3D Lidar image was subjected to the Region Proposal Network (RPN) to obtain the feature image, which has five continuous convolution and activation operations. The activation function using here is rectified linear unit (ReLU). On each pixel of the feature image, 20 anchor regions were generated to predict the bounding box of the stem area. In this experiment, four areas (12, 72, 142, and 592) and five ratios (0.13, 0.51, 0.84, and 1.3, 8) corresponding to the anchors were obtained according to the size and shape of the target stem area in the training sample. For each anchor, we calculated the probability of whether it was foreground or background. If the anchor had the largest degree of overlap with the target object or the overlap degree was greater than 0.7, it was marked as the foreground sample. If the overlap was less than 0.3, then it was the negative sample. The other ambiguous anchors, whose overlap were around 0.5 were not involved in the training. Moreover, for each anchor, we calculated the parameters that each anchor should be shifted and scaled based on its position offset from the ground truth bounding box of the target object. Therefore, the loss function of Faster R-CNN consisted of classification and regression loss, and the detailed information can be found in the work of Ren et al. (2015). Faster R-CNN has achieved extraordinary results in 2D images detection, which is promising to learn the ability of detecting stem in 2D images compressed from 3D points. The whole process of using Faster R-CNN and regional growth algorithm was shown in **Figure 3** and will be described in the flowing sections.

#### Training Faster R-CNN Model to Learn Stem Location in 2D Images

The ultimate goal of the training was to enable Faster R-CNN to find the target area on a compressed 2D image. The 10784 training samples generated from 3D points were sent into the Faster R-CNN model. The “end-to-end” CNN was built based on the Caffe deep learning framework (Jia et al., 2014), which learned the ability to detect the location of a stem given a maize with complicated background. We trained the network with the base learning rate of 0.01, momentum of 0.9 (represent the weight of the last gradient update), and weight decay of 0.0005 to avoid overfitting. In each epoch, we used stochastic gradient descent to optimize the loss function. The model was trained until the classification and segmentation loss were both satisfied (generally both smaller than 0.01).

#### Testing Faster R-CNN Model to Predict Stem Location in 2D Images

The general idea of using the Faster R-CNN to detect target in 3D points was that the model can detect target region with the 2D views from 3D Lidar image. The testing process contained four main steps. For each testing data, the 3D points were firstly sliced into different 3D window with a field size of 1.024 m × 1.024 m, and a depth of 0.016 m. The field in the x and z direction was ensured to cover a maize, the depth of 0.016 m was used to detect stem but with less background. As the deeper the depth, the more background will be included in the 2D images. Secondly, the sliced points were used to generate 2D images. Thirdly, these 2D images were used to predict the location of stem. Only the predicted results with more than 90% prediction confidence were kept. Finally, the location of the slice unit and four coordinates of the predicted anchor of the stem in each compressed image can be recorded, which will be used to map the 3D points. To reduce the undetected stem, we sliced the 3D points of each site from 32 different directions, and repeated the above testing process. For the well-scanned maize with complete stem, the Faster R-CNN can detect the stem from all directions. Among the detected stems of the same maize, we only kept the one whose prediction confidence was the best. For the incomplete maize with irregular shape, the Faster R-CNN can detect the stem from some specific directions, and the result was kept.

#### Mapping Stem From 2D Images to 3D Points and Realizing Individual Maize Segmentation

Each 2D anchor of the predicted image provide the x and z value of the stem location. The slice window of the image provided the y value of the bounding box. Using the x, y, and z value, we can map the 2D anchor into its corresponding 3D space, which covered the original 3D stem points. The stem points were treated as seed points to grow each maize from bottom to up using a the CSP regional growth method proposed by Tao et al. (2015). The CSP method was inspired by the metabolic ecology theory and has been proved with good accuracy in forests. The CSP method included three parts: points normalization, trunk detection and diameter at breast height (DBH) estimation, and finally segmentation. In this study, we only adopted the segmentation part. In this part, 3D transporting distances of each point to different trunks (D_v_) were calculated and scaled by the DBH as formula (1). The shortest scaled distance ($D_{v}^{N}$) of each point to different trunks determined which trunk the point should belong to.

(1) $$D_{v}^{N}=D_{v}/DBH^{2/3}$$

#### Assessment of Segmentation Accuracy

The segmentation result was evaluated at individual maize level for all the three different sites. If a maize was labeled as class A and segmented as class A, it was true positive (TP); if a maize was labeled as class A but was not segmented (allocated to another class), it was false negative (FN); if a maize did not exist but was segmented, it was false positive (FP). We expected higher TP, lower FN, and lower FP to get higher accuracy. Moreover, the recall (*r*), precision (*p*), and F-score (*F*) for each

(2) $$\begin{array}{c} r=\frac{\text{TP}}{\text{TP+FN}}\\ p=\frac{\text{TP}}{\text{TP+FP}}\\ F=2^{*}\frac{r^{*}p}{r+p} \end{array}$$

site were calculated using the following equations (Goutte and Gaussier, 2005).

Moreover, for the truly segmented individual maize, we compared the height with manually segmented height using correlation of coefficients (*R*^2^) and root-mean-squared error (RMSE).

## Results

### Segmentation Results of the Three Sites

**FIGURE 4:**
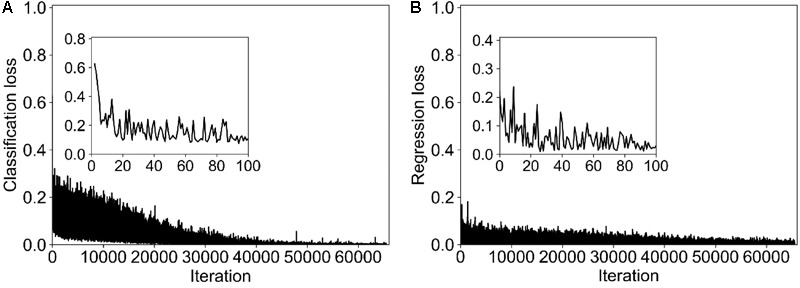
The training loss of the Faster R-CNN for detecting stem of maize in deep images. **(A)** Loss of classification; **(B)** loss of segmentation.

In this study, we trained 65000 epochs totally. Although the loss of classification and regression was not smooth enough, the overall decline trend was obvious (**Figure 4**). The loss declined mainly in the first 100 epochs. The final classification and segmentation loss was around 0.0005 and 0.003, respectively, which represents the small error between the predicted results and the corresponding ground truths. The total training time was around 1 h on a PC with intel i7-7700k CPU, 16 GB RAM, and a NIVIDA GTX 1070 GPU.

**FIGURE 5:**
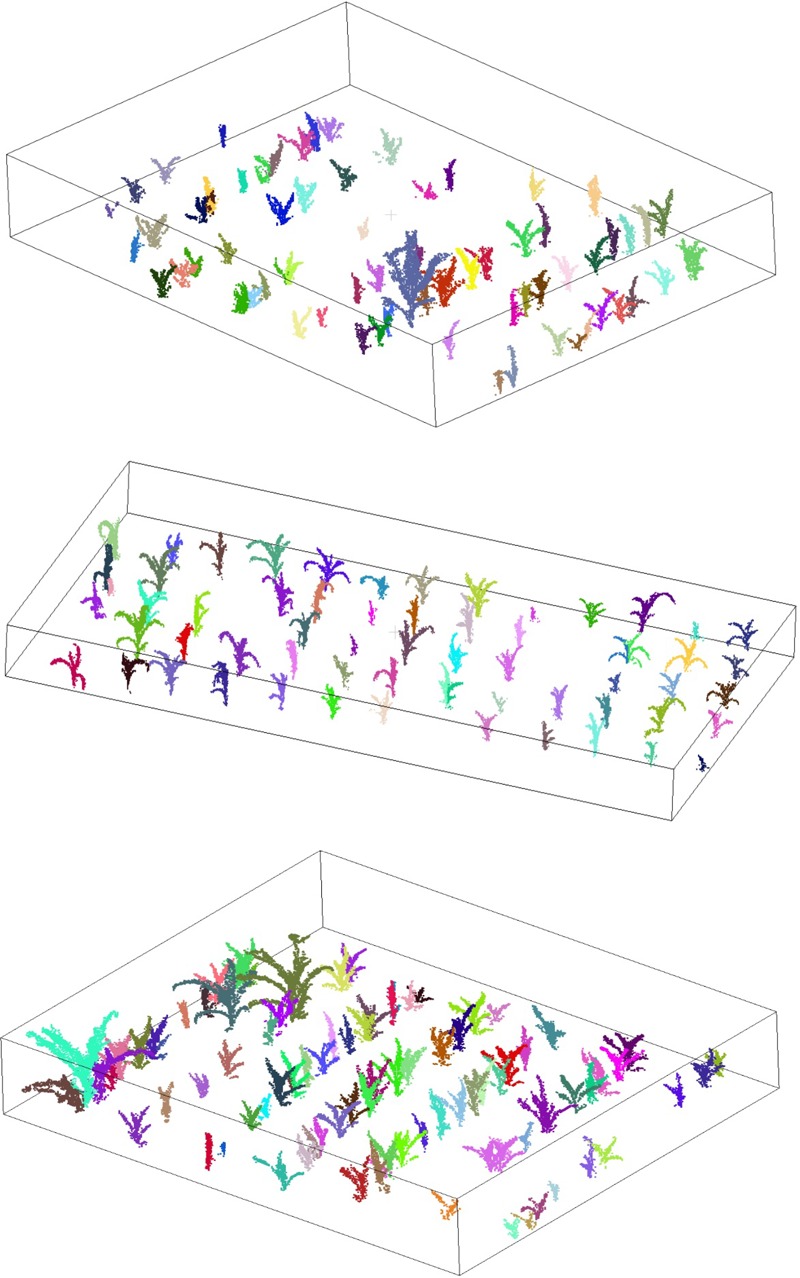
The segmentation results of sparse (top), moderate (middle), and dense (bottom) testing sites. The segmented individual maize are represented by unique colors.

The segmentation results of the three test sites were all good (**Figure 5**). The overall values of r, p, and F were 0.93, 0.94, and 0.94, respectively. For the sparse site, the values of r, p, and F were 0.95, 0.93, and 0.94, respectively. For the moderate site, the values of r, p, and F were 0.93, 0.97, and 0.95, respectively. For the dense site, the values of r, p, and F were 0.93, 0.94, and 0.94, respectively (**Table 3**). These segmented accuracies were almost the same despite of different planting density when scanned in 32 different directions. We visually compared the segmentation results by varying the number of scanning directions and found 32 was the best. The testing time of the sparse, moderate, and dense site are around 6, 8, and 13 min, separately.

**Table 3:** The accuracy assessments of the individual maize segmentation on the three testing datasets with different planting density.

	TP	FP	FN	R	P	F
Sparse	59	4	3	0.95	0.93	0.94
Moderate	66	2	5	0.93	0.97	0.95
Dense	81	6	7	0.92	0.93	0.93
Overall	206	12	15	0.93	0.94	0.94

### Height Prediction Accuracy Using Segmented Results

**FIGURE 6:**
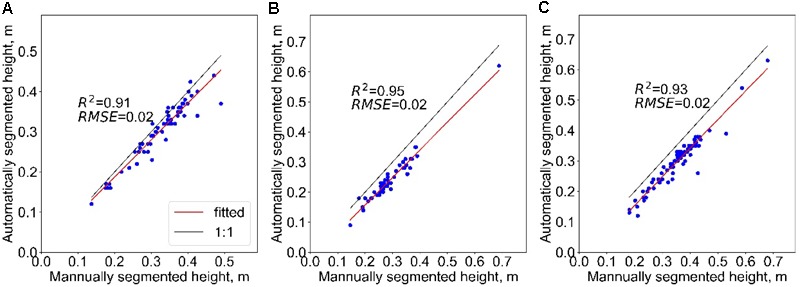
The correlation of the individual maize height of automatically and manually segmented in maize group with planting density of sparse **(A)**, moderate **(B)**, and dense **(C)**.

The *R*^2^ of the height between the automatically segmented and the manually segmented maize were all higher than 0.9, and RMSE all equalled to 0.02 m. The best result appeared at the site of moderate density, which seems to have no relationship with the planting density. In addition, the heights of maize of the automatically segmented were lower than the manually segmented results in all the three sites. The mean underestimation of sparse, moderate, and dense sites was 0.02, 0.05, and 0.06 m, separately (**Figure 6**).

## Discussion

### Segmentation Results

The segmentation results all showed high accuracy from sparse to dense planting density, which benefited from the high accuracy of stem detection by Faster R-CNN. There might be three main reasons for this. Firstly, each site was tested from 32 different directions, and the joint result contributed to the high accuracy. Secondly, the slice depth of the 3D window was only 0.016 m, which was smaller than the interval of any two adjacent maize samples at any of the three sites. In this situation, the planting density is not a determining factor and the isolated stem is easy to be detected. Thirdly, points within the 3D window with a depth of 0.016 m is enough to keep the shape of stem when compressed into 2D images. Even the 3D points are incomplete and noisy, the Faster R-CNN can detect the stem in the image based on the regional and global background information. Theoretically, the 0.016 m depth can well-segmented maize group whose interval was bigger than the threshold of 0.016 m. If the plant density became denser, the scanning depth may need to be slightly smaller. In reality, the planting interval is usually larger than this threshold, which means the method can be applied to perform segmentation in fields with different planting densities. Moreover, as the method segmenting maize from bottom to up, the overlap of leaf has little influence on the detection of stem.

This method has made up for the deficiency of image-based method through using the spatial information of 3D point cloud. However, there were still some shortcomings. Firstly, the proposed method was only tested with young maize plants in the elongated stage with a maximum height of around 0.7 m. The effectiveness of the proposed method for segmenting maize plants in mature stage still needs to be further studied. Nevertheless, we believed that the proposed method has the potential to handle mature maize segmentation. The main reasons can be concluded from two aspects: (1) the stem location is fixed after seedling and the stem width does not change much after elongated stage, which might be detected with a similar accuracy of the current datasets; and (2) the proposed method used a fixed 3D window size with a depth of 0.016 m covering only a piece of the background information, which will not change too much even in the mature stage. Moreover, the integrity of individual maize points derived from the regional growth method might be influenced by the heavily intercepted leaves, but it might have limited influence on the extracting phenotype parameters, such as maize height. Secondly, if stem was completely missing in the scanned data, the network cannot detect the individual maize, which cannot further grow to an individual stem. Thirdly, if there was a leaf of maize drops to the ground and looks like a stem from the side, the leaf may be falsely detected as a stem, and further grow to a false positive individual maize.

### Height Prediction Accuracy

The high correlation (*R*^2^ > 0.9) of the automatically predicted and manually measured plant height enabled breeders to conduct the height-related phenotyping experiments in a high throughput way, which showed great potential in the field environment. HTP information of plant height can reflect the plant biomass and stress, etc. (Madec et al., 2017), which can further be used to assist gene selection and accelerate crop breeding. However, some limitations still exist. The predicted maize height of all three sites were all lower than the ground truth. The reason might be that we removed ground points automatically with a global threshold when mapping 2D anchor to 3D points. Currently, almost all the available filtering algorithms cannot fully remove ground points, especially when the ground points have a depth of few centimeters as well as micro-topography. Unremoved ground points may affect the speed and accuracy of the regional growth algorithm, which is undesirable. Therefore, we removed the ground point with a global threshold of 0.1 m, which means the points will be removed if they were at the neighbor of 0.1 m of the lowest ground points in each small area. However, the mean underestimation of the three sites were all less than 0.1 m. The reason is the maize was planted on top of the ridge in the field, the removed ground points often contain less stem points. In the following work, we plan to develop new filtering algorithm to remove ground points, like deep learning based method (Hu and Yuan, 2016), which can help this task to get more accurate tree height information.

## Conclusion

In this study, we demonstrated the combination of deep learning and regional growth methods to segment individual maize with terrestrial Lidar scanned 3D points. A total of 10784 images compressed from 337 individual maize samples were used to train the Faster R-CNN to learn the ability of detecting stems. Three sites of the same growing stage with different planting densities were used to test the stem detection ability. These tested stems were further mapped into 3D points. The results showed that the Faster R-CNN based method is powerful in detecting stem anchor in 2D views from 3D Lidar images. The regional growth method can accurately segment the individual maize with the detected stem seed points. Although there are some false positive and false negative errors, the higher accuracy with r, p, and F of more than 90% can significantly reduce the workload to get 100% correct result by purely manual methods. Overall, the segmented height of maize was highly correlated to the manually measured value, demonstrating our method can obtain accurate height of individual maize. Because the seed points were detected by the Faster R-CNN and the regional growth method algorithm had no parameter, the proposed method is non-parametric, and has the possibility to be applied in other field conditions.

## Author Contributions

SJ, QG, and YS: conceived the idea and proposed the method. FW, SP, DW, and YJ: contributed to the preparation of equipment and acquisition of data. SJ and SG: wrote the code and tested the method. SJ, QG, SC, and YS: interpreted results. SJ wrote the paper. YS, TH, JL, and WL: revised the paper. All authors read and approved the final manuscript.

## Conflict of Interest Statement

The authors declare that the research was conducted in the absence of any commercial or financial relationships that could be construed as a potential conflict of interest.

## Footnotes

1 http://www.worldometers.info/world-population/
